# Assessment of satisfaction of attendees of healthcare centers in Jordan with community pharmacy services of pharmacies they usually use

**DOI:** 10.1371/journal.pone.0305991

**Published:** 2024-07-22

**Authors:** Ghaith M. Al-Taani, Nehad M. Ayoub

**Affiliations:** 1 Department of Clinical Pharmacy and Pharmacy Practice, Faculty of Pharmacy, Yarmouk University, Irbid, Jordan; 2 Department of Clinical Pharmacy, Faculty of Pharmacy, Jordan University of Science and Technology, Irbid, Jordan; Bolton Clarke Research Institute, AUSTRALIA

## Abstract

Before extending the range of services provided, maximizing the usefulness of current procedures within community pharmacy settings is needed, as the scope of pharmacy services is evolving in different dimensions. The present study aims to assess the degree of factors affecting the satisfaction of traditional community pharmacy services using population data collected from patients attending academic and public healthcare centers in Jordan. A validated, pretested, and adapted survey instrument has been utilized to assess the satisfaction of contemporary services delivered by community pharmacists in different dimensions. Linear regression analysis evaluated the predictors associated with higher total satisfaction scores with community pharmacy services. The present study included 642 patients attending healthcare centers. Different dimension scores, such as explanation and consideration, scored similarly, with values ranging from 64.5% - 69.7% of the maximum possible score. The mean total scale score was 67.2% of the total possible scores. Using the linear regression analysis, respondents who were satisfied with their treatment plans were likely to have higher satisfaction with community pharmacy services. The increased number of prescription medications and increased age were associated with lower satisfaction with community pharmacy services. Results indicated that healthcare policymakers might be confident in the services within the community pharmacy setting; however, there is always room for more robust quality control activities.

## Introduction

Pharmacists contribute to public health through direct patient care activities, including dispensing essential medicines and other professional services, such as pharmaceutical care [1–3]. The satisfaction of pharmacy services is a popular metric to assess the success of both traditional and innovative services [4]. Satisfaction is a multi-dimensional concept concerned with individual subjective perception of the quality and efficacy of pharmacy services [5]. It offers valuable information about whether the service, often new, is well-received by customers and can suggest modifications or further developments [6]. Satisfaction can be viewed as the degree of positive feeling about the service by evaluating what they experienced [7]. In pharmacy practice research, it is expressed as the humanistic component of the economic, clinical, and humanistic outcomes [8]. Such humanistic assessment is patient-reported outcomes that ensure the service is viable and can be continued. This provides valuable information for the pharmacist, overhead professional bodies, and healthcare system decision-makers. A well-recognized framework for outcome assessments in pharmacy practice in a systematic way is the structure, process, and outcome framework [9]. Nowadays, it is imperative to assess these humanistic-based outcomes for any health service, including patient satisfaction, and pay attention to the traditional clinical-based outcomes. Ensuring patient satisfaction is relevant to patient-centered care [10] provides the availability of pharmaceutical services [11]. It increases the likelihood of achieving the desired treatment targets, translating to better quality of medical practices [12].

The pharmacist’s skill and competence are essential to eliciting satisfaction with the community pharmacy services. [13]. Patient satisfaction with community pharmacy services is usually perceived according to how the service is provided, particularly regarding the general communication approach and soft skills [14]. The communication process provided by the pharmacist is essential to enhanced satisfaction, as it provides information about the medicines to ensure correct use and best outcomes, expresses empathy, is active listening, and ensures patient-centered care [15]. In addition, keeping pace with newer developments within the pharmacy sector mandates expanding pharmaceutical services [16]. Professional communication tools that the pharmacist can employ emphasize the rapport and trust within the professional relationship between the pharmacist and patient and would be associated with a positive impact on the patients’ loyalty and satisfaction [17–19]. Other factors related to service delivery associated with satisfaction arise from how the offered services are provided regarding convenient access to the pharmacy services via extended working hours, increased timeliness in service, and technology integration [20–22]. Furthermore, waiting times had the inverse relationship with satisfaction level [23].

In recent years, there has been a movement towards increased patient engagement and centeredness in pharmacy practice; such an approach individualizes the patient-pharmacist intervention and incorporates the patient input through their communicated concerns and preferences within the clinical decision-making [14]. Besides, to ensure optimal satisfaction with the service, it is imperative that the pharmacist applies a caring approach and invests the encounter time to use pharmaceutical and clinical knowledge to achieve the best patient outcomes [1, 24]. Such professional attributes have been included as the intended learning outcome within the newly established Center for the Advancement of Pharmacy Education (CAPE) for pharmaceutical education. When pharmacy services are pointed out as poor quality, this might lead to certain patient behaviors, such as decreased patient retention and poor compliance [1, 2]. It is known that consistent and accurate dispensing is a critical factor for patient satisfaction, while errors in dispensing affect the perception of the quality of service.

In Jordan, two studies have assessed satisfaction with community pharmacy services using online surveys. These studies focused on community pharmacy’s business orientation and perception [25, 26]. As competency is important given that the scope of pharmacy services is evolving in different dimensions, the present study aims to assess the degree of and factors affecting satisfaction with community pharmacy services in a sample of patients attending public and academic healthcare centers from different regions in Jordan. The present study allows for assessing key multifaceted services offered routinely by community pharmacies using a face-to-face approach.

## Methods

The present study was conducted in healthcare centers, both academic and public, in Amman, the national capital of Jordan, and Irbid, a large city in the north of Jordan, from June 2021 to July 2022.

### Data collection

Using convenient sampling, adult patients attending academic and public healthcare centers in different geographical locations in Jordan were recruited for this study. The included healthcare centers were the Jordan University of Science and Technology healthcare center in Irbid, an academic healthcare center in Irbid, Jordan that is affiliated with Jordan University of Science and Technology, and additional public ministry of health-based healthcare centers, including Al Husun Healthcare center in Irbid, Shafa Badran healthcare center, and Abu Nusair healthcare center in Amman. These centers provide primary healthcare; in these centers, the patients are assessed by physicians, supported by nursing staff, and dispensed with acute and long-term medicines by pharmacists. These healthcare centers have an emergency department and laboratory as well.

Patients were eligible to participate in this study if they had been prescribed at least one drug for a chronic condition (prescription or supplement). Patients who cannot give informed consent were excluded from the study. A trained research assistant screened the potential participants, approached those who fit the criteria, and explained the study to them while waiting for their scheduled medical appointments. The research assistant highlighted voluntary participation and the insurance of confidentiality. Those who agreed to take part in this study were requested to provide written informed consent and to complete a validated, pretested survey using a face-to-face approach. The use of a face-to-face survey method allows the completion of the study tool whilst the participants are present in the healthcare center. This will allow professionals to clarify any ambiguous items and allow for obtaining responses from those with low digital literacy or who do not have access to the internet. The research assistant supported the participants in completing the survey. Using an online sample size calculator, Raosoft (available at: http://www.raosoft.com/samplesize.html), and considering a 5% margin of error, 95% confidence interval, and 50% response distribution, the present study aimed to recruit 385 participants. As the recruitment rate achieved was high, the recruitment of additional participants was sought to address the limitations of the convenience sampling method and to improve the generalizability of the findings. The protocol of this study was approved by the following ethics committees: the Ministry of Health Research Ethics Committee (MOH/REC/2022/1), the institutional review board of Jordan University of Science and Technology (57/139/2021), and Yarmouk University Institutional Review Board (IRB/2021/6).

### Instrument

A survey instrument has been utilized to assess the satisfaction of contemporary services delivered by the community pharmacists that patients attending academic and public healthcare centers usually use. The survey instrument included two parts: 1. respondents’ background details, and 2. an adapted Arabic-translated form of the validated Satisfaction with Pharmacy Services Questionnaire [27, 28]. A standardized Arabic translation process was carried out. A forward translation to Arabic was carried out and the Arabic version translated was subject to independent backward translation to English to ensure an accurate translation of the ideas and concepts of the survey, and any issues within the translation process were addressed.

The respondents’ details included nine items about gender, age, marital status, educational level, occupation, monthly income, health insurance, number of prescription drugs used, and satisfaction with the treatment plan.

The Satisfaction with Pharmacy Services Questionnaire includes 29 items that belong to five dimensions: explanation, consideration, technical competence, physical attributes, and general care. These dimensions coin the traditional day-to-day services of the community pharmacist. In the consideration dimension, questions related to the pharmacist spending the necessary time with the patient and filling prescriptions promptly. The explanation dimension related to patient counseling activities carried out by the community pharmacist, whereas the technical competence was related to the thoroughness of the pharmacist’s actions, along with accurate and correct dispensing of prescriptions. The physical attributes are related to pharmacy premises. Lastly, the general dimension is related to the overall satisfaction with the pharmacy services. Each item in every dimension has five responses: strongly agree, agree, not sure, disagree, and strongly disagree. Each dimension included favorably worded items and unfavorably worded items, as well as matching pairs. The instrument’s validity was established using principal component and item analysis to confirm different construct measures within the dimensions developed [27]. Furthermore, the instrument was reliable, with coefficient alpha ranging from 0.6958 to 0.8442 for the other scales [28]. The translated version of the survey tool was reviewed by experts in this type of research, including four faculty members from the School of Pharmacy at Yarmouk University with clinical experience and an MSc and PhD degree, as well as an experienced pharmacist with an interest in the innovative medication therapy management within the community pharmacy setting, to ensure the face and content validity of the survey. Additionally, the survey was pilot-distributed to 10 potential participants to ensure the items’ clarity and the distribution logistics appropriateness. Based on the comments from the faculty members and the pilot group, minor modifications were made to the survey.

### Data analysis

Standard statistical methodologies were used to assess the satisfaction with community pharmacy services. The Statistical Package for Social Sciences (SPSS) was used for the analysis. Frequencies, percentages, and means were used to summarize the data. Linear regression analysis assessed the predictors associated with higher total satisfaction scores with community pharmacy services. Statistical significance was set at P≤0.05.

## Results

### Description of study participants

The present study included 642 patients attending healthcare centers in Jordan. Approximately seventy percent of the respondents were females. About one-third (34.1%) of the respondents were aged between 18 years and 30 years old. About sixty percent of the respondents were married, and almost one-half of them had a BSc degree. Most of the respondents were unemployed, one-fifth of the respondents were students, and 11.9% were retired. 14.0% of the respondents had a monthly income of more than 1000 JD. Most (91.1%) respondents were medically insured, and 81.3% were prescribed three or fewer prescription drugs. About two-thirds of the respondents are satisfied and/or highly satisfied with their current treatment plan. Full details about the background characteristics of study respondents are summarized in Table 1.

**Table 1:** Characteristics of the study population.

Variable		Frequency	Percent
Gender	Male	192	29.9
	Female	450	70.1
Age (Years)	18–30	216	34.1
	31–40	71	11.2
	41–50	93	14.7
	51–65	187	29.5
	More than 65	66	10.4
Marital status	Single	235	36.7
	Married	406	63.3
Educational level	Uneducated	29	4.6
	Secondary	167	26.3
	Diploma	92	14.5
	Bsc	311	48.9
	Graduate studies	37	5.8
Occupation	Unemployed	277	42.9
	Medical/health occupation	35	5.4
	Non-medical occupation	118	18.3
	Student	139	21.5
	Retired	77	11.9
Monthly income (JD)	Less than 500	237	36.7
	500–1000	223	34.6
	More than 1000	90	14.0
	Prefer not to answer	95	14.7
Health insurance	Insured	565	91.1
	Uninsured	55	8.9
Number of prescription drugs	0–3	526	81.3
	4–6	100	15.5
	7–15	21	3.2
Satisfaction with treatment plan	Highly unsatisfied	33	5.3
	Unsatisfied	57	9.1
	Neutral	126	20.2
	Satisfied	371	59.5
	Highly satisfied	37	5.9

### Satisfaction with community pharmacy services

Table 2 summarizes the results for the individual statements indicating satisfaction with community pharmacy services. In the explanation dimension, 20.1% of the respondents strongly agreed, “If I have a question about my prescription, the pharmacist is always available to help me”. While in the consideration dimension, 24% of the respondents strongly agreed with the statement, “The pharmacist spends as much time as is necessary with me”. Regarding technical competence, 19.4% of the respondents strongly agreed with “The pharmacist is always thorough”. Regarding physical attributes, 19.3% of the respondents strongly agreed, "The pharmacy area is as clean as any medical office”. In the general dimension, 18.8% of the respondents strongly agreed with “I am very satisfied with the pharmacy services”.

**Table 2:** Individual statements indicating satisfaction with community pharmacy services.

Dimension / Statement	Strongly agree n (%)	Agree n (%)	No opinion (%)	Disagree n (%)	Strongly disagree n (%)
*Explanation*					
The pharmacist usually explains the possible side effects that a new medication may cause.	122 (19.6)	311 (49.8)	77 (12.3)	77 (12.3)	37 (5.9)
If I have a question about my prescription, the pharmacist is always available to help me.	126 (20.1)	325 (51.9)	94 (15.0)	57 (9.1)	24 (3.8)
The pharmacist knows how to explain things in a way that I understand.	121 (19.3)	296 (47.2)	138 (22.0)	41 (6.5)	31 (4.9)
When I get a prescription filled, the pharmacist makes sure that I understand how to take the medication.	115 (18.3)	270 (43.0)	141 (22.5)	80 (12.7)	22 (3.5)
The pharmacist hardly ever explains what my medication does.	53 (8.5)	130 (20.8)	117 (18.7)	302 (48.3)	23 (3.7)
I sometimes have trouble understanding what the pharmacist means when he or she talks to me about my medication.	41 (6.5)	80 (12.8)	157 (25.1)	317 (50.6)	31 (5.0)
The pharmacist often does not tell how to take my prescription medication.	49 (7.8)	114 (18.2)	155 (24.8)	279 (44.6)	29 (4.6)
*Consideration*					
The pharmacist spends as much time as is necessary with me.	150 (24.0)	319 (51.0)	73 (11.7)	59 (9.4)	25 (4.0)
My prescriptions are always filled promptly.	109 (17.5)	278 (44.6)	154 (24.7)	61 (9.8)	22 (3.5)
The pharmacy staff seems to have a genuine interest in me as a person.	104 (16.6)	212 (33.9)	175 (28.0)	110 (17.6)	24 (3.8)
The pharmacy staff are always courteous and respectful.	122 (19.5)	320 (51.2)	118 (18.9)	49 (7.8)	16 (2.6)
Sometimes the pharmacist does not spend enough time with me.	51 (8.2)	110 (17.6)	137 (21.9)	294 (47.0)	33 (5.3)
The pharmacy staff should be more friendly.	52 (8.4)	79 (12.7)	121 (19.5)	338 (54.5)	30 (4.8)
The pharmacist should do more to keep people from having problems with their medications.	108 (17.3)	277 (44.2)	137 (21.9)	82 (13.1)	22 (3.5)
I usually have to wait a long time when I get a prescription filled.	80 (12.8)	161 (25.7)	150 (24.0)	203 (32.4)	32 (5.1)
*Technical competence*					
I am confident that the pharmacist dispenses all prescriptions correctly	117 (18.7)	246 (39.2)	170 (27.1)	74 (11.8)	20 (3.2)
The pharmacist is always thorough.	121 (19.4)	269 (43.0)	165 (26.4)	55 (8.8)	15 (2.4)
The pharmacist is not as thorough as he/she should be.	39 (6.3)	108 (17.4)	194 (31.2)	247 (39.7)	34 (5.5)
I sometimes wonder about the accuracy of the prescriptions that the pharmacist dispenses.	43 (6.9)	101 (16.1)	167 (26.7)	287 (45.8)	28 (4.5)
*Physical attributes*					
The pharmacy area is as clean as any medical office.	121 (19.3)	336 (53.7)	115 (18.4)	39 (6.2)	15 (2.4)
The pharmacy has a comfortable waiting area.	82 (13.1)	185 (29.6)	145 (23.2)	156 (25.0)	57 (9.1)
The pharmacy staff always looks professional.	105 (16.8)	270 (43.2)	178 (28.5)	46 (7.4)	26 (4.2)
There is no place where I can have a private conversation with the pharmacist.	80 (12.8)	199 (31.7)	129 (20.6)	182 (29.0)	37 (5.9)
There are so many distractions in the pharmacy area that you often can’t get good service.	53 (8.5)	112 (17.9)	159 (25.4)	279 (44.6)	23 (3.7)
The prescription area is too cluttered with signs and merchandise.	47 (7.5)	80 (12.8)	168 (26.9)	299 (47.8)	31 (5.0)
*General*					
The pharmacy services that I receive are just about perfect.	103 (16.5)	227 (36.3)	206 (33.0)	64 (10.2)	25 (4.0)
I am very satisfied with the pharmacy services.	117 (18.8)	271 (43.6)	156 (25.1)	59 (9.5)	19 (3.1)
There are things about the pharmacy services that could be better.	109 (17.4)	228 (36.4)	170 (27.1)	96 (15.3)	24 (3.8)
I have some complaints about the pharmacy services.	45(7.2)	122 (19.5)	133 (21.2)	302 (48.2)	24 (3.8)

The total score for different scales and the total scale score are summarized in Table 3. The results revealed similar scores concerning the dimension scores and total scale scores. For example, the explanation domain scored 69.7% as the average score expressed as a percentage of the maximum possible score, and the consideration domain average score was 66.7%. The average total scale score was 67.2% of the maximum possible score; this is expressed as 97.5 out of 145, the maximum possible score.

**Table 3:** Summative score for the scale dimension and total score of satisfaction with community pharmacy services.

Scale	Number of items	Maximum possible score	Average score (out of maximum possible score)	Standard deviation	Average score (percentage)
Explanation domain	7	35.0	24.4	4.7	69.7
Consideration domain	8	40.0	26.6	4.5	66.4
Technical competence domain	4	20.0	13.7	2.5	68.7
Physical attributes domain	10	30.0	19.9	3.4	66.3
General domain	4	20.0	12.9	2.3	64.5
Total Score	37	145.0	97.5	14.9	67.2

### Factors affecting satisfaction with community pharmacy services

Table 4 describes the details of the linear regression analysis for patient-related independent factors associated with the dependent variable, which is the higher total satisfaction scores with community pharmacy services. Respondents who were satisfied with the treatment plan were more likely to have higher satisfaction with community pharmacy services with a positive magnitude of association with a score of 2.841, which indicates that with each unit of satisfaction with the treatment plan, the satisfaction with community pharmacy services is increased by 2.841 folds, keeping other predictors constant. The increased number of prescription medications and increased age is associated with lower satisfaction with community pharmacy services, in which with each unit of the increased number of prescription medications and age, the total satisfaction score is decreased by 0.743 and 0.147, respectively, keeping other predictors constant.

**Table 4:** Patient-related predictors of higher total satisfaction score with community pharmacy services using linear regression analysis.

Variable	B (standard error)	P value	95% confidence interval or B
Constant	65.049 (2.006)	<0.001	61.109–68.988
Satisfaction with treatment plan	2.841 (433)	<0.001	1.991–3.691
Number of prescription medications	-0.743 (0.229)	0.001	-1.194 –-0.292
Age	-0.147 (0.023)	<0.001	-.192 –-0.102

## Discussion

The purpose of the current study is to assess the satisfaction of patients attending public and academic healthcare centers with the community pharmacy services they usually use. They were collected at this time to align with the expansion of pharmacists’ professional services and the increase in the number of pharmacy providers in Jordan. The validated instrument used in the present study was designed to echo the conventional services provided by community pharmacies, and it included elements of both pharmacies and pharmacists. Our analysis revealed that patients are generally satisfied with the services they receive from the pharmacies they usually use. Patient satisfaction with the treatment plan predicted greater satisfaction with community pharmacy services. Patients who are prescribed many medications and those who are older are less satisfied with the services provided by community pharmacies. These population-level statistics provide insight into how the public views the overall standard of the services offered in the community pharmacy setting.

Satisfaction with community pharmacy services is an indicator reflecting the degree of quality of services [29]. This study assessed the satisfaction of community pharmacy services using a multidimensional approach. The total satisfaction score was equal to 67.2% of the total possible score in the present study, encompassing input from four dimensions. One must keep in mind that the assessment of the specific dimension score can be more reasonable. Overall, the scores for different dimensions were approximately 60% of the total possible score. These results reveal an acceptable acceptance of contemporary pharmacy services from population data. This can strengthen the vital role of the pharmacist as the leading player in satisfaction with community pharmacy services. Pharmacists provide pivotal services that improve public health, including high accessibility and provision of patient counseling, which is expected to increase satisfaction with pharmacy services [29, 30]. The reported results were limited to the traditional and typical roles of the pharmacist; it is anticipated that the community pharmacy would, in due time, carry out other specialized professional activities, such as medication therapy management [31]. It is clear that if we want to expand the pharmacist’s roles, traditional activities must be mastered before taking up new roles. A review article highlighted high pharmacy service satisfaction in published studies [32]. High satisfaction has been reported with community pharmacy services internationally, and if dissatisfaction occurs, it might be related to lax in dispensing and responding to patient requests [33]. High accessibility to pharmacy services and medications and the presence of cognitive services have been reported as factors that enhance patient satisfaction with community pharmacy services [22].

The pharmacist is a crucial accessible healthcare professional in providing patient education. Patients and the general public appreciate pharmacists’ advice regarding medications, supplements, and disease, an important policy issue of interest to pharmacists, overhead professional bodies, and healthcare policymakers. Appropriate provision of patient education services ensures therapeutic outcomes [34]. Interestingly, about 60% of the patients surveyed in the present study were satisfied with the explanation. This highlights that pharmacists provide routine explanations regarding medicines to the public in a way they can understand. The pharmacist is best suited to provide this pivotal role and other advice related to a healthy lifestyle [35]. Research evidence has highlighted that the appropriate and clear communication provided by pharmacists is more likely to lead to satisfaction with the community pharmacy services [36], and this directly reflects on the critical therapeutic outcomes [34]. A related piece of work highlighted a higher prevalence of satisfaction with counseling by community pharmacists, which equaled 72% [37]. This figure is close to that reported in the present study. Other researchers have reported poor satisfaction with counseling services owing to the limited time invested in counseling; acknowledging this factor and carefully considering other factors would be a means to improve pharmacy services [29].

Other services under the umbrella of consideration were provided in the present study in about 60% of the participant responses. This highlights that pharmacists spend adequate time with their patients. With an increased workload, pharmacists might not be able to spend much time with their patients, and indeed, lack of time can hinder the provision of patient counseling services by the community pharmacist [29]. It is worth mentioning that having spent time with patients in the community pharmacy is associated with better healthcare coordination and would reflect on patient satisfaction [38]. Satisfaction with community pharmacy services is related to the time spent in counseling and dispensing, and if this has not taken place, dissatisfaction with community pharmacy services would be evident [29, 39, 40].

The importance of pharmacist knowledge, skills, and competence cannot be underemphasized in the satisfaction with community pharmacy services. In the present study, about two-thirds of the participants were satisfied with the skills and competence of the pharmacists working in the community pharmacy they routinely use. Such a skill mix starts within the degree program, and then the cumulative work experience and continuous education effort are major contributors [41–43]. Skills such as creative thinking and problem-solving are linked with specific subject courses in the pharmacy curriculum [44]. In line with this, pharmacists should be prepared for the ever-changing nature of pharmacy services in the era of online information for pharmacists and patients, and the convenience of smartphones in pockets [45, 46]. Poor awareness of the full range of services provided by community pharmacists can be evident, particularly cognitive services, which might relate to poor competency of pharmacists, low contact with patients, and unsatisfactory pharmacist knowledge [24]. Such trends might impact the satisfaction with community pharmacy services. Patients should not view the pharmacist as a dispenser. In previous research, it has been highlighted that about three-quarters of the patients surveyed were satisfied with the role of the pharmacist in providing advice on the proper use of their medicines, and about half of the responders were satisfied with the key role of the pharmacist in answering the queries they have relating to their medicines. These views stem directly from the pharmacist’s competency [36].

The last dimension studied of satisfaction with community pharmacy services was the physical attributes of the community pharmacy premises. The present study participants were satisfied in about 60% of the cases with the premises of the community pharmacy, highlighting their usefulness in providing accessible healthcare. This satisfaction is, however, temporary as enhanced community pharmacy services are not currently provided within the community pharmacy. Further adjustment to the pharmacy layout may be required if enhanced pharmacy services such as medication therapy management are to be delivered. If such changes are not made, there will be doubts about the ability of community pharmacists to expand their services [47]. Satisfaction with community pharmacy services can be linked to the pharmacy premises, including being useful in providing medication information, maintaining privacy, providing easy-to-find products by the patients, and having a dedicated consultation room [48].

Better services and communication from the community pharmacist are major contributors to patient satisfaction in the community pharmacy [36, 49]. Our findings revealed that patients who were satisfied with their treatment plans were more likely to be satisfied with the community pharmacy services. Such patients might be more engaged in communication and participation in treatment plans, reflecting their higher satisfaction levels in pharmacy services. Such shared decision-making would help overall satisfaction with community pharmacy services [50]. Additionally, if the treatment plan is free from errors and misunderstandings, patients are more likely to be satisfied with their plans and the provided pharmacy services [51, 52]. Empowering patients with their treatment plan would improve patient satisfaction [53].

A higher number of medications implies challenges for medication management for the patients, particularly due to increased treatment complexity, leading to confusion about the proper information on their medicines and increased likelihood of adverse effects [54, 55]. This would challenge community pharmacy services, particularly patients’ with a lack of access to medical records and reduced health literacy [56, 57]. Indeed, a higher number of medications predicted poor satisfaction with community pharmacy services in the present study. Furthermore, polypharmacy reflects an increased number of comorbid conditions and medications, with some drugs having specific properties hindering the pharmacist’s role in patient care [58]. Notably, in certain situations, polypharmacy might be justifiable and appropriate and would be needed for the optimal treatment of patients [59]. On the contrary, this can increase the need for more cognitive pharmacy services [60].

Results from this study indicated that increased age is associated with low patient satisfaction with pharmacy services. To provide effective pharmacy services, special consideration should be given to elderly patients. Consideration should be paid to communication issues, cognitive impairment, and poor health literacy in older people, which might cause miscommunication and reduced satisfaction with pharmacy services [61, 62]. Elderly patients also had challenges in adopting new technologies and digital communication platforms, which would be integrated into the pharmacy services and could overwhelm this group of patients, leading to poor satisfaction with pharmacy services [63]. Despite this, these results are not concordant with other published research, highlighting that increased age is associated with increased satisfaction with community pharmacy services [64, 65], which might be linked to the characteristics of the population studied, such as the level of education, the settings and nature of the pharmacy services provided to patients, and their comorbidities.

The present study assessed the satisfaction of community pharmacy settings with traditional services, particularly the dispensing of medications, patient education, and pharmacy premises. A logical extension to the present study is to assess recipients’ acceptability, satisfaction, and willingness to pay for additional professional services from community pharmacies. A complementary project that can be carried out in the community pharmacy setting is to assess the accessibility and affordability of medicines from community pharmacists using patient-level data. Also, the present study suggests the need for future research concerning the indicators for community pharmacy services, to ensure rigorous quality of services. The present study suggests future research into the facilitators and barriers that affect the satisfaction of community pharmacy services. Improvement of the skill mix of pharmacists might be sought in terms of credentialing professional activities to be carried out by pharmacists.

The present study was conducted using a validated scale that safeguards against potential bias and informs rigorous assessment of the degree of satisfaction of the public with the community pharmacy settings. The interpretation of present study findings might be interpreted with caution owing to the sampling approach, as convenient sampling was carried out with this limitation and increased the generalizability of the findings. The convenient sampling from primary healthcare centers might affect the type of population the present study is generalizable to; however, the impact of this issue is minimal due to both of the settings being primary points of contact for patients, and it is envisioned that those who interact with community pharmacists would have access to the primary healthcare center. A possible limitation might be difficulties understanding some questions the participants asked. To minimize this issue, a face-to-face approach was utilized to distribute the questionnaire, and the research assistant trained on this project was readily available to clarify any misunderstandings. The patients might be answering questions because they target specific favorable responses, termed social desirability bias. Such limitations might not be highly pronounced in the present study owing to the anonymous survey distribution.

## Conclusions

The present study attempted to assess the degree of satisfaction with community pharmacy services by surveying patients attending healthcare centers in Jordan using validated, pretested, and well-adapted instruments. The findings highlighted that traditional pharmacy services are well-received, which can facilitate expanding pharmacy services. Patients who were satisfied with community pharmacy service were more likely to be satisfied with their treatment plans. Increased number of medications and increased age were factors associated with low satisfaction with the community pharmacy services. Results recommended that healthcare policy makers be confident in the services within the community pharmacy setting. However, there is always room for more robust quality control activities.
